# Supplementary material for: Partitioning of the initial catalytic steps of leucyl-tRNA synthetase is driven by an active site peptide-plane flip
Source: Commun Biol. 2022 Aug 29;5:883. doi: 10.1038/s42003-022-03825-8 (PMC9424281; doi:10.1038/s42003-022-03825-8)
Supplement: Supplementary file 2 — Supplementary Information [file 42003_2022_3825_MOESM2_ESM.pdf]

Supplementary Information

Partitioning of the initial catalytic steps of leucyl-tRNA synthetase is driven by an active site peptide-plane flip

Luping Pang[1,2,3], Vladimir Zanki[4], Sergei V. Strelkov[1], Arthur Van Aerschot[2,*], Ita Gruic-Sovulj[4,*] and Stephen D. Weeks[1,5,*]

[1] Biocrystallography, Department of Pharmaceutical and Pharmacological Sciences, KU Leuven, Herestraat 49 – Box 822, 3000 Leuven, Belgium

[2] Medicinal Chemistry, Rega Institute for Medical Research, Department of Pharmaceutical and Pharmacological Sciences, KU Leuven, Herestraat 49 – Box 1041, 3000 Leuven, Belgium

[3] Research Center of Basic Medicine, Academy of Medical Sciences, College of Medicine, Zhengzhou University, Zhengzhou, Henan, 450001, China

[4] Department of Chemistry, Faculty of Science, University of Zagreb, Horvatovac 102a, 10000 Zagreb, Croatia

[5] Pledge Therapeutics, Gaston Geenslaan 1, 3001 Leuven, Belgium

*Corresponding authors
Stephen D. Weeks: Pledge Therapeutics, Gaston Geenslaan 1, 3001 Leuven, Belgium
E-mail: sweeks@pledge-tx.com

Ita Gruic-Sovulj: Department of Chemistry, Faculty of Science, University of Zagreb, Horvatovac 102a, 10000 Zagreb, Croatia
E-mail: gruic@chem.pmf.hr

Arthur Van Aerschot: Medicinal Chemistry, Rega Institute for Medical Research, KU Leuven, Herestraat 49 box 1041, 3000 Leuven, Belgium
E-mail: arthur.vanaerschot@kuleuven.be

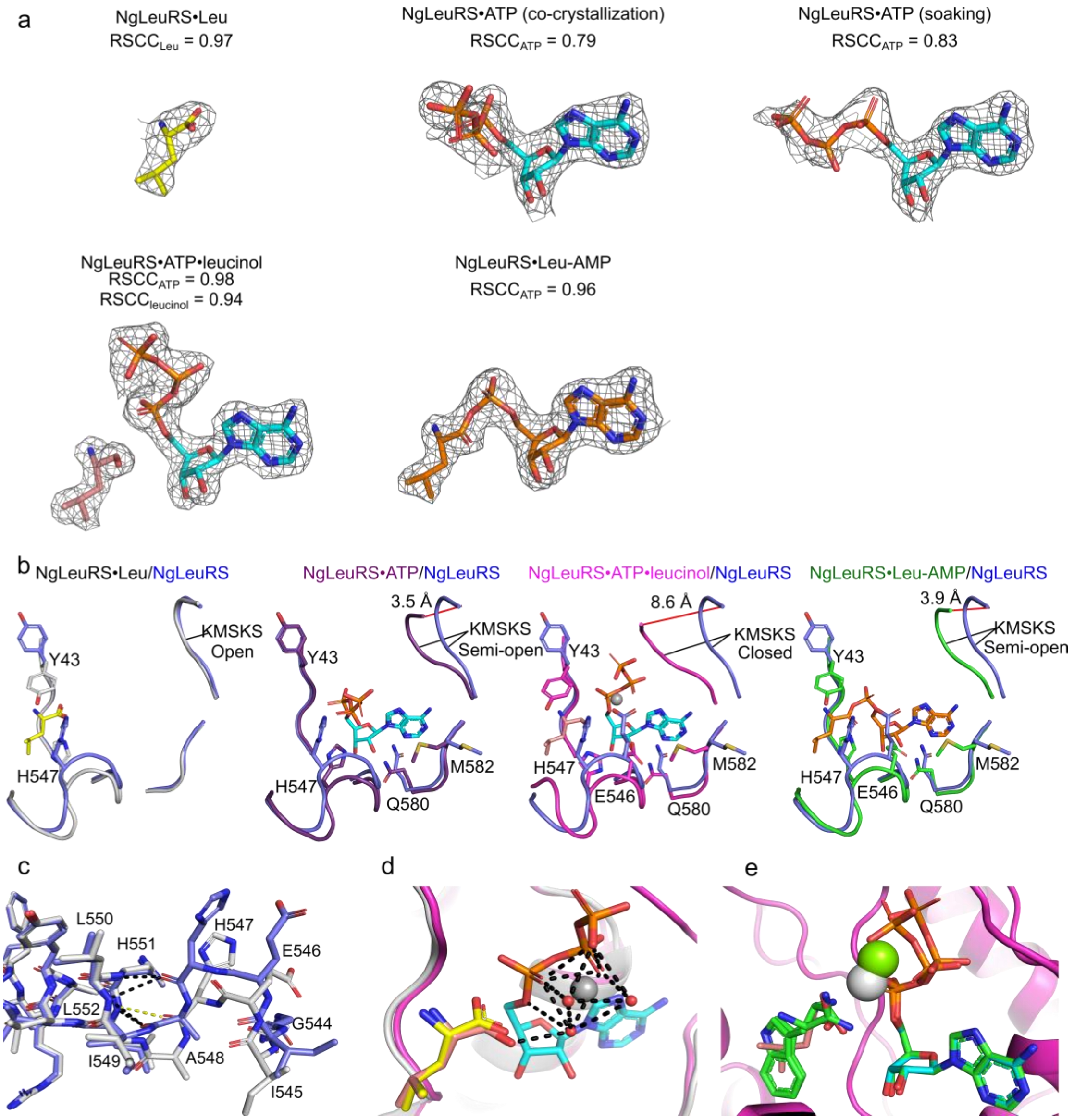

Supplementary Fig. 1 | NgLeuRS-substrate complexes. a, Omit maps (grey mesh) of the ligands in the different solved LeuRS-substrate complexes calculated using Phenix.Polder[1].The reported real-space correlation coefficient (RSCC) of each ligand shows that is well bound in the active site of NgLeuRS. Two different ATP binding conformations were obtained by co-crystallization and soaking methods. Omit maps of ligands are countered in a range of 3.5 - 5 σ. **b**, Comparison of the active site conformation of the ligand-free and different substrate-bound NgLeuRS structures. The protein backbones are shown as ribbon representations, specific protein residues and substrates are shown as sticks. NgLeuRS, NgLeuRS·Leu, NgLeuRS·ATP (obtained by co-crystallization), NgLeuRS·ATP·leucinol and NgLeuRS·Leu-AMP are colored in slate, grey, purple, magenta and green, respectively. The residues with different rotamers and conformational changes of the KMSKS loop are highlighted. Individual structures were superposed using residues encompassing the aminoacylation, CP2, Zn2 and anti-codon binding domains. **c**, Superposition of the N-terminus of L550-containing α-helix

(residues H^{547}-E^{565}) in the apo and Leu-bound structures. The H-bonds in the apo structure are shown as black dashed lines while the H-bond in NgLeuRS·Leu is shown as a yellow dashed line. **d**, Comparison of ligand-bound conformations in the active site of the superposed structures of NgLeuRS·Leu and NgLeuRS·ATP·leucinol. The magnesium ion is shown as a grey sphere with surrounding water molecules shown as small red spheres. **e**, Structural alignment of NgLeuRS·ATP·leucinol with *Geobacillus stearothermophilus* TrpRS·ATP·tryptophanamide based on the adenine of ATP. The carbon atoms of ATP, leucinol and the magnesium ion in the former structure are colored in cyan, salmon and grey, respectively, while in the TrpRS·ATP·tryptophanamide structure, the corresponding carbon atoms of ATP, tryptophanamide and magnesium ion are all green.

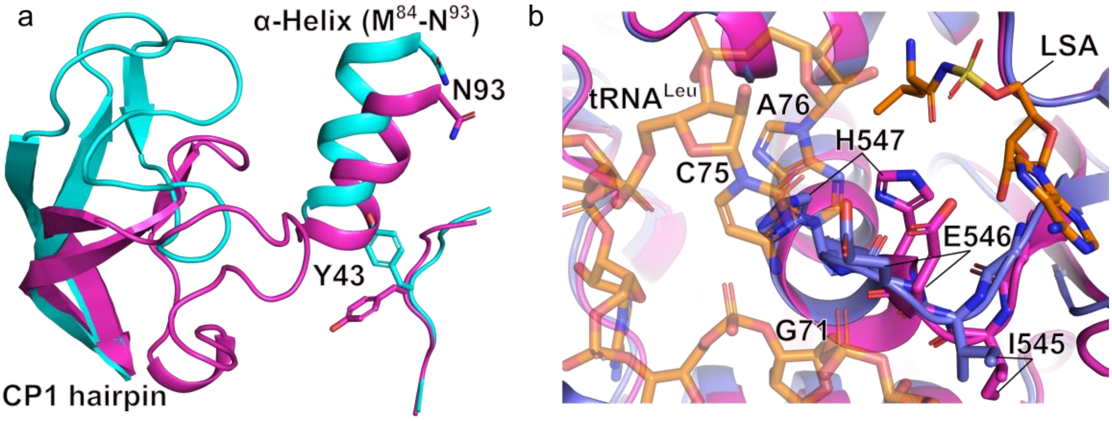

Supplementary Fig. 2 | Additional conformational changes observed during NgLeuRS catalysis. **a**, Closure of the CP1 hairpin is dependent on a shift of α-helix (M^{84}-N^{93}). Conformational changes in the vicinity of the CP1 hairpin that are associated with the formation of the pre-transition state are shown by superposition of NgLeuRS·ATP·leucinol (magenta) with NgLeuRS·ATP (cyan) structures. The binding of ʟ-leucinol (or Leu) induces a rotamer switch of Y43 lid residue. This rearrangement is essential in allowing the α-helix (M^{84}-N^{93}) to move in concert with the CP1 hairpin during formation of the pre-transition state. The protein structures are shown as cartoon representations while Y43 and N93 are shown as sticks. For clarity the ligands have been omitted. **b**, The relaxed position of the priming loop is incompatible with positioning of the 3'-end of the $tRNA^{Leu}$ substrate in the aminoacylation site. Structural superposition of the ternary complex of EcLeuRS·$tRNA^{Leu}$·LSA (PDB ID: 4AQ7, magenta) and NgLeuRS (slate). LSA is the abbreviation of leucyl-sulfamoyl adenosine, an non-hydrolysable analog of Leu-AMP. Once the activated Leu is transferred to $tRNA^{Leu}$, the priming loop can relax to the original state seen in the apo enzyme as a loss of the interactions with the amino acid and AMP byproduct. The superposition clearly shows that this relaxed state would result in steric clashes between the side chains of H547, E546 of the priming loop and A76, C75, G71 of the $tRNA^{Leu}$ and suggests that the priming loop may have a role in the translocation of the charged $tRNA^{Leu}$ from the aminoacylation site to the catalytic region of the editing domain.

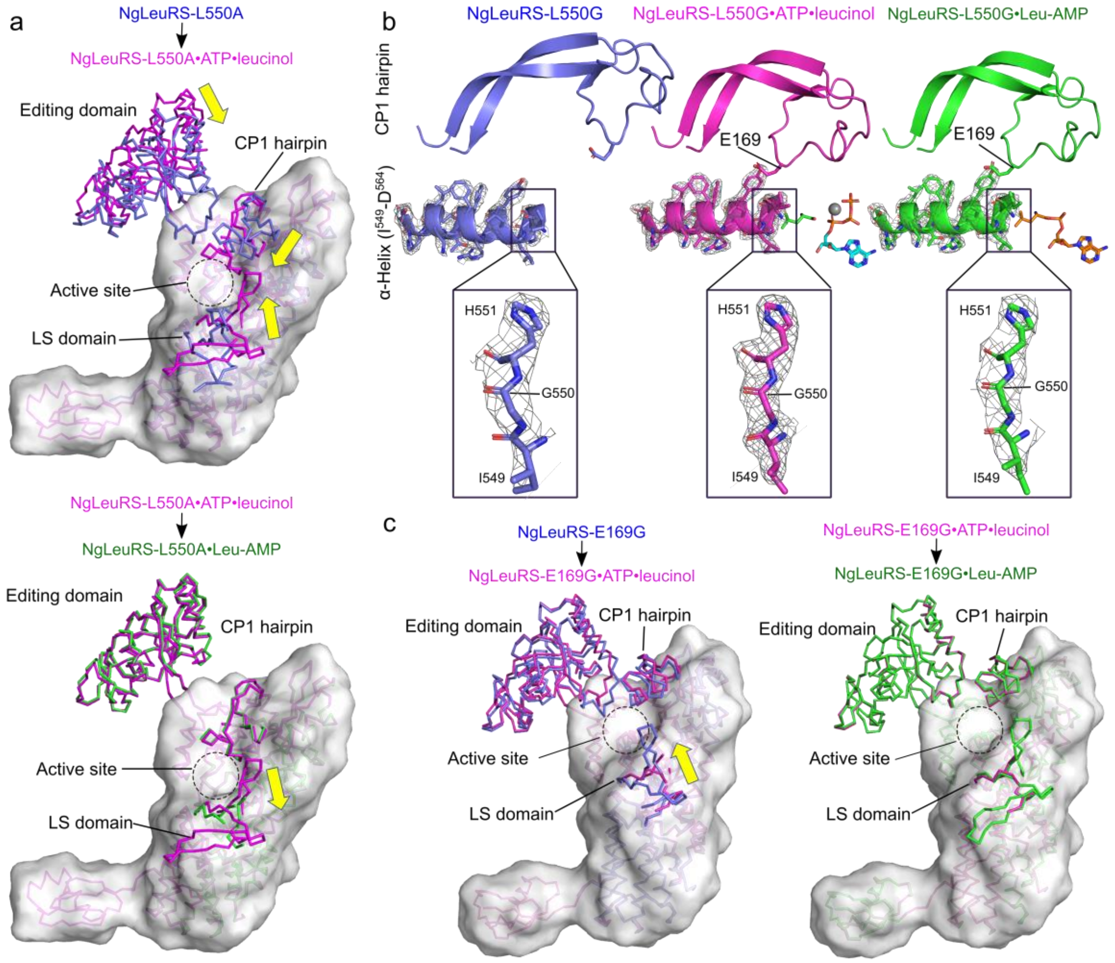

Supplementary Fig. 3 | Structural comparison of different catalytic states of NgLeuRS mutants. a, Top, ribbon representation of the superposition of the ternary structure of NgLeuRS-L550A·ATP·leucinol (magenta) with NgLeuRS-L550A (slate). Bottom, superposition of the ternary structure of NgLeuRS-L550A·ATP·leucinol (magenta) with NgLeuRS-L550A·Leu-AMP (green). In both panels the aminoacylation domain, CP2, Zn2, ACBD and CTD of the NgLeuRS-L550A·ATP·leucinol structure are additionally shown as a surface representation. **b**, Top panels, the relative position of the CP1 hairpin (cartoon backbone representation) to the L550G-containing α-helix (cartoon and stick representation) in different catalytic states of NgLeuRS-L550G mutant. Ligands are shown as stick representations while the magnesium ion is shown as a grey sphere. Bottom, zoom of the G550 region. 2Fo-Fc electron density maps (grey mesh) are countered at 1.5 σ. **c**, Left, ribbon representation of the superposition of the ternary structure NgLeuRS-E169G·ATP·leucinol (magenta) with NgLeuRS-E169G (slate). Right, superposition of the ternary structure NgLeuRS-E169G·ATP·leucinol (magenta) with NgLeuRS-E169G·Leu-AMP (green). In both panels, the aminoacylation domain, CP2, Zn2, ACBD and CTD of the NgLeuRS-E169G·ATP·leucinol structure are additionally shown as a surface representation.

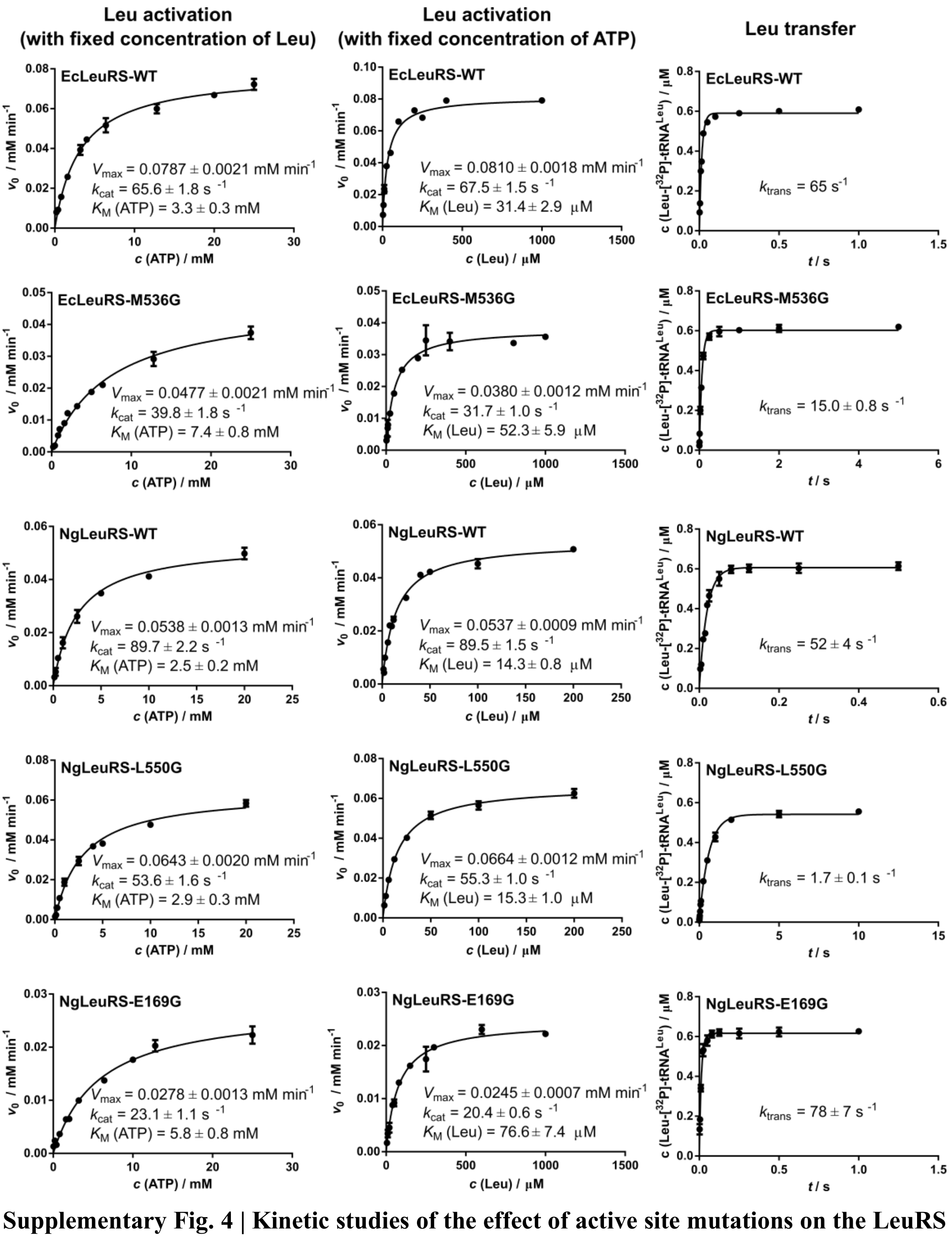

Supplementary Fig. 4 | Kinetic studies of the effect of active site mutations on the LeuRS catalysis. The first two columns show the measurements of the Leu activation step where either the Leu concentration is fixed at 1 mM (left) or the ATP is fixed at 25 mM (middle). The last column shows the measurements of Leu transfer step. For all NgLeuRS constructs and the EcLeuRS-M536G mutant, experiments were performed in triplicate with error bars shown. For the WT EcLeuRS the transfer experiment was only performed once, but has being extensively characterized previously, yielding similar results[2,3]. All data for these plots are provided in the Supplementary Data 2 file.

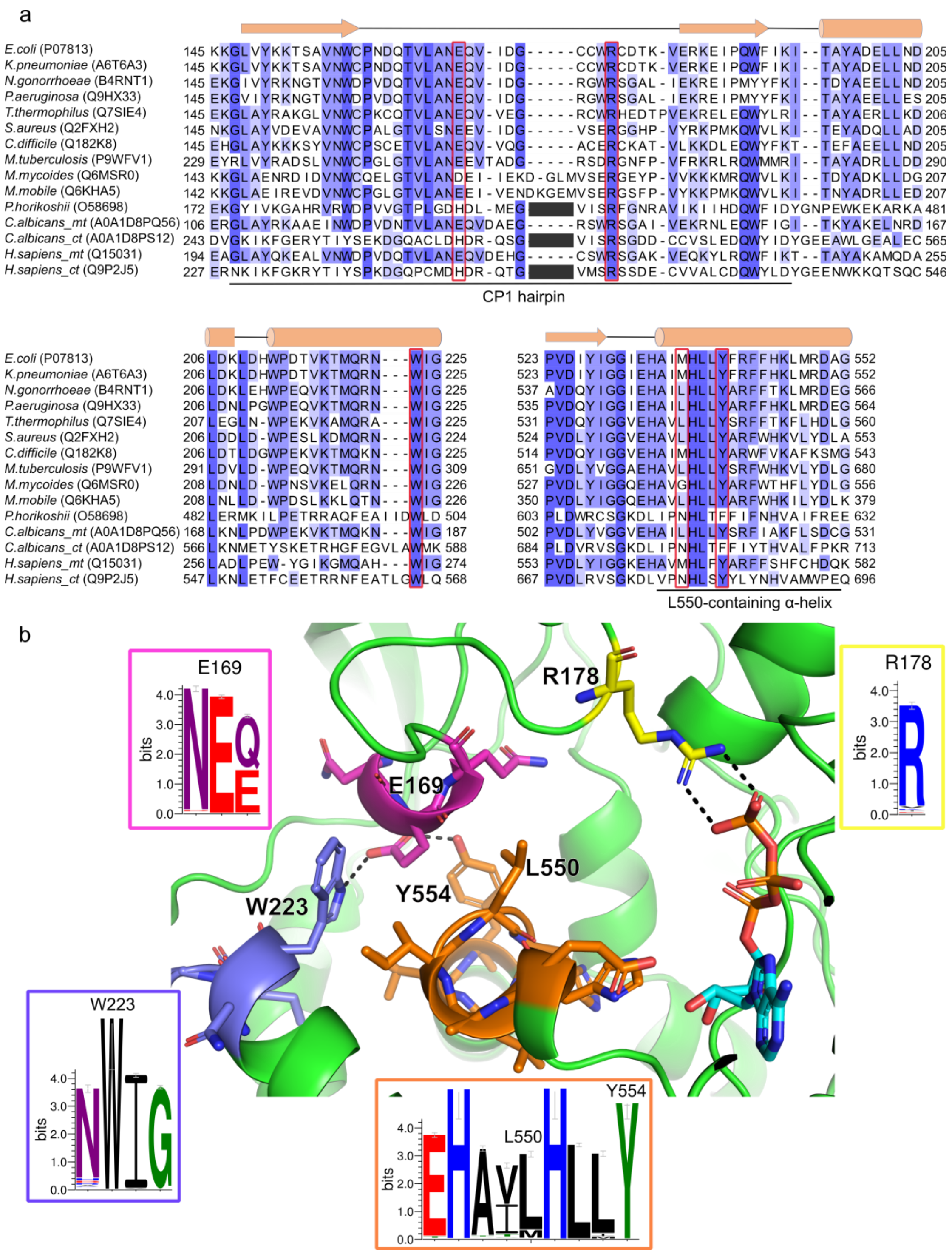

Supplementary Fig. 5 | Structural and sequence conservation of LeuRS. a, Sequence alignment of LeuRSs from different species. Residues showing greater than 40% conservation are highlighted whereby the shading corresponds to the sequence identity. The equivalent residues corresponding to E169, R178, W223, L550 and Y554 in NgLeuRS are highly conserved and shown in red boxes. In archaeal and eukaryotic cytosolic LeuRSs the editing domain is found inserted in the middle of CP1 hairpin primary sequence, and for simplicity is represented as a black filled box in the alignment. The secondary structure of the NgLeuRS is

mapped onto the alignment, where the orange cylinder represents an α-helix and the orange arrows represent a β-strand. **b**, Sequence conservation of the essential elements that stabilize the fully closed pre-transition state of bacterial LeuRSs. The protein backbone is shown as a cartoon representation while important residues and the ATP substrate are shown as sticks. For clarity L-leucinol and the catalytic magnesium ion are not shown. Essential conserved sequence elements are represented as a WebLogo[4] and are mapped onto the structure according to the box outline.

Supplementary References

1. Liebschner, D. *et al.* Polder maps: improving OMIT maps by excluding bulk solvent. *Acta Crystallogr D Struct Biol* **73**, 148–157 (2017).
2. Cvetesic, N., Perona, J. J. & Gruic-Sovulj, I. Kinetic partitioning between synthetic and editing pathways in class I aminoacyl-tRNA synthetases occurs at both pre-transfer and post-transfer hydrolytic steps. *J Biol Chem* **287**, 25381–25394 (2012).
3. Cvetesic, N., Palencia, A., Halasz, I., Cusack, S. & Gruic-Sovulj, I. The physiological target for LeuRS translational quality control is norvaline. *EMBO J* **33**, 1639–1653 (2014).
4. Crooks, G. E., Hon, G., Chandonia, J.-M. & Brenner, S. E. WebLogo: a sequence logo generator. *Genome Res* **14**, 1188–1190 (2004).
