# Supplementary material for: Partitioning of the initial catalytic steps of leucyl-tRNA synthetase is driven by an active site peptide-plane flip
Source: Commun Biol. 2022 Aug 29;5:883. doi: 10.1038/s42003-022-03825-8 (PMC9424281; doi:10.1038/s42003-022-03825-8)
Supplement: Supplementary file 3 — Description of Additional Supplementary Files [file 42003_2022_3825_MOESM3_ESM.pdf]

Description of Additional Supplementary Files

File name: Supplementary Data 1
Description: X-ray crystallographic data reduction and refinement statistics.

File name: Supplementary Data 2
Description: The source data behind the graphs in the paper.
