# Supplementary material for: Partitioning of the initial catalytic steps of leucyl-tRNA synthetase is driven by an active site peptide-plane flip
Source: Commun Biol. 2022 Aug 29;5:883. doi: 10.1038/s42003-022-03825-8 (PMC9424281; doi:10.1038/s42003-022-03825-8)
Supplement: Supplementary file 6 — Reporting Summary [file 42003_2022_3825_MOESM6_ESM.pdf]

nature portfolio

Corresponding author(s): Stephen D. Weeks, Ita Gruic-Sovulj, Arthur Van Aerschot

Last updated by author(s): Jul 13, 2022

Reporting Summary

Nature Portfolio wishes to improve the reproducibility of the work that we publish. This form provides structure for consistency and transparency in reporting. For further information on Nature Portfolio policies, see our Editorial Policies and the Editorial Policy Checklist.

Statistics

For all statistical analyses, confirm that the following items are present in the figure legend, table legend, main text, or Methods section.

n/a	Confirmed	
☐	☒	The exact sample size (*n*) for each experimental group/condition, given as a discrete number and unit of measurement
☐	☒	A statement on whether measurements were taken from distinct samples or whether the same sample was measured repeatedly
☒	☐	The statistical test(s) used AND whether they are one- or two-sided<br>*Only common tests should be described solely by name; describe more complex techniques in the Methods section.*
☒	☐	A description of all covariates tested
☒	☐	A description of any assumptions or corrections, such as tests of normality and adjustment for multiple comparisons
☒	☐	A full description of the statistical parameters including central tendency (e.g. means) or other basic estimates (e.g. regression coefficient) AND variation (e.g. standard deviation) or associated estimates of uncertainty (e.g. confidence intervals)
☒	☐	For null hypothesis testing, the test statistic (e.g. *F*, *t*, *r*) with confidence intervals, effect sizes, degrees of freedom and *P* value noted<br>*Give P values as exact values whenever suitable.*
☒	☐	For Bayesian analysis, information on the choice of priors and Markov chain Monte Carlo settings
☒	☐	For hierarchical and complex designs, identification of the appropriate level for tests and full reporting of outcomes
☒	☐	Estimates of effect sizes (e.g. Cohen's *d*, Pearson's *r*), indicating how they were calculated

Our web collection on statistics for biologists contains articles on many of the points above.

Software and code

Policy information about availability of computer code

Data collection	mxCuBE
Data analysis	Pymol 2.0.4, Coot 0.9.4, Phenix 1.19.2-4158, Autoproc 1.0.5, Jalview 2.11.2.0, Schrödinger 2021-2, GraphPad Prism 6.0

For manuscripts utilizing custom algorithms or software that are central to the research but not yet described in published literature, software must be made available to editors and reviewers. We strongly encourage code deposition in a community repository (e.g. GitHub). See the Nature Portfolio guidelines for submitting code & software for further information.

Data

Policy information about availability of data

All manuscripts must include a data availability statement. This statement should provide the following information, where applicable:

- Accession codes, unique identifiers, or web links for publicly available datasets
- A description of any restrictions on data availability
- For clinical datasets or third party data, please ensure that the statement adheres to our policy

Atomic coordinates and structure factors have been deposited in the protein data bank (PDB) with the following accession codes: 7NU4 (NgLeuRS), 7NU5 (NgLeuRS in complex Leu), 7NU6 (NgLeuRS in complex with ATP in conformation 1), 7NU7 (NgLeuRS in complex with ATP in conformation 2), 7NU8 (NgLeuRS in complex with Leu-AMP), 7NU9 (NgLeuRS in complex with ATP and l-leucinol), 7NTY (NgLeuRS-L550A), 7NTZ (NgLeuRS-L550A in complex with Leu-AMP), 7NU0 (NgLeuRS-L550A in complex with ATP and l-leucinol), 7NUA (NgLeuRS-L550G), 7NUB (NgLeuRS-L550G in complex with Leu-AMP), 7NUC (NgLeuRS-L550G in complex with ATP and l-leucinol), 7NU1 (NgLeuRS-E169G),), 7NU2 (NgLeuRS-E169G in complex with Leu-AMP), 7NU3 (NgLeuRS-E169G in complex with ATP and l-leucinol).

Field-specific reporting

Please select the one below that is the best fit for your research. If you are not sure, read the appropriate sections before making your selection.

☒ Life sciences ☐ Behavioural & social sciences ☐ Ecological, evolutionary & environmental sciences

For a reference copy of the document with all sections, see nature.com/documents/nr-reporting-summary-flat.pdf

Life sciences study design

All studies must disclose on these points even when the disclosure is negative.

Sample size	Sample size was determined on the basis of a large number of previous studies using similar methods and dealing with similar proteins.
Data exclusions	For structure refinement 2000 reflections, or a maximun of 5% of the data, were excluded for R-free calculations.
Replication	Biochemical assays were performed in triplicate using protein from the same purification batch.
Randomization	No randomisation was preformed.
Blinding	Investigators were not blinded to the sample allocations because all the samples and conditions were predetermined and analysed using the same methods.

Reporting for specific materials, systems and methods

We require information from authors about some types of materials, experimental systems and methods used in many studies. Here, indicate whether each material, system or method listed is relevant to your study. If you are not sure if a list item applies to your research, read the appropriate section before selecting a response.

Materials & experimental systems

n/a	Involved in the study
☒	☐ Antibodies
☒	☐ Eukaryotic cell lines
☒	☐ Palaeontology and archaeology
☒	☐ Animals and other organisms
☒	☐ Human research participants
☒	☐ Clinical data
☒	☐ Dual use research of concern

Methods

n/a	Involved in the study
☒	☐ ChIP-seq
☒	☐ Flow cytometry
☒	☐ MRI-based neuroimaging
